# Tuberculous meningitis initially manifesting as acute areflexic paraparesis: A case report

**DOI:** 10.1002/ccr3.7698

**Published:** 2023-07-18

**Authors:** Balintona Reynaldo, Memon Noor Illahi, Tarab Iqbal, Sidra M. Nayyar, Abdulqadir J. Nashwan

**Affiliations:** ^1^ Department of Medicine, Hazm Mebaireek General Hospital Hamad Medical Corporation Doha Qatar; ^2^ Department of Nursing, Hazm Mebaireek General Hospital Hamad Medical Corporation Doha Qatar

**Keywords:** acute Paraparesis, spinal Arachnoiditis, tuberculous meningitis, tuberculous Radiculomyelitis

## Abstract

**Key Clinical Message:**

TBM has a very high rate of adverse sequelae if not treated immediately. Diagnosing can be challenging due to overlapping symptoms with other disease processes, and diagnostic tests are often inconclusive.

**Abstract:**

A 20‐year‐old man experienced progressive paraplegia and urinary retention. After extensive laboratory and imaging evaluation for tuberculous meningitis and alternative diagnoses, spinal MRI showed features suggestive of arachnoiditis. He was treated empirically with anti‐tuberculosis drugs and corticosteroids. This led to significant improvement and eventual recovery.

## Graphical abstract

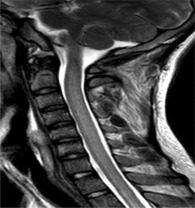

## 1 INTRODUCTION

Tuberculosis (TB) is one of the deadliest infectious illnesses that causes approximately 1.5 million deaths each year, and it continues to be a major global health problem.^1^ Tuberculous meningitis (TBM), which is a form of extrapulmonary TB, is relatively rare but remains highly fatal, often leading to death within 8 weeks if prompt treatment is not initiated.^2^ TBM commonly presents in sub‐acute form with a prodrome of fever, malaise, and headache progressing to a meningitic phase after a period of approximately 2–3 weeks. However, some patients have atypical symptoms and they may initially present with focal neurological signs such as hemiparesis, convulsions, or cranial nerve palsies before signs of meningitis set in.^2^ Confirming the diagnosis of TBM can be challenging, as traditional tests such as cerebrospinal fluid (CSF) cultures and TB cultures, and smears for Acid‐Fast Bacilli (AFB) may produce inconclusive results due to low to moderate sensitivity of these methods as compared to newer molecular methods such as PCR. Therefore, it remains crucial to make a presumptive diagnosis based on a high clinical suspicion for TBM (such as a history of exposure to TB, alcohol intake, elderly, immunocompromised, epidemiological setting) initiating empiric treatment promptly even without awaiting laboratory confirmation.^2^, ^3^ This report aims to enhance clinicians' understanding of the atypical presentation of TBM, focusing on a patient exhibiting clinical signs of radiculomyelitis, MRI evidence of spinal arachnoiditis, and a negative pathogen test result.

## 2 CASE PRESENTATION

A 20‐year‐old male was brought to our Emergency Department in November 2022 with complaints of difficulty walking, associated with an inability to pass urine and confusion. It was his fourth visit in a week. He had presented earlier with complaints of fever, headache, and suprapubic pain and was treated with oral antibiotics, on suspicion of Urinary Tract Infection (UTI). His last trip to India was 3 months ago. On admission, he was afebrile, anxious with power in both lower limbs 3/5 (according to Manual Medical Strength Testing by the Medical Research Council), and absent deep tendon reflexes (DTR). Initial blood tests, including complete blood count (CBC), metabolic panel, blood culture, carbohydrate antigen reactive protein (CRP), urinalysis, and computerized tomography (CT) of the brain, was reported normal. Lumbar Puncture (LP) was done, which showed clear fluid with leukocytosis and lymphocytic predominance, high protein, and low glucose with low CSF/serum glucose ratio (Table 1). CSF bacterial culture, a viral panel screen, and Cryptococcus neoformans antigen were also negative. All comprehensive testing for TB, with CSF AFB smears, PCR, and culture, including blood quantiferon tests were negative. Additionally, extensive testing for alternative pathologies like human immunodeficiency virus (HIV), Brucellosis, Guillain Barre Syndrome (GBS), and virology panels showed negative results. Initial magnetic resonance imaging (MRI) of the brain and spine showed bilateral extensive leptomeningeal enhancement throughout the cerebral and cerebellar hemispheres.

**TABLE 1:** Laboratory investigations.

	Results	Normal value
Complete blood count		
White blood cell	10.6	4–10 × 10^3uL
Neutrophils	78.5	40%–60%
Lymphocytes	7.3	20%–40%
Monocytes	8.4	2%–8%
Eosinophils	4.9	1%–4%
Basophils	0.9	0.5%–1%
Hemoglobin	13.9	13–17 gm/dL
Platelet	308	150–410 × 10^3uL
Clinical chemistry		
Urea	3.6	2.7–7.8 mmol/L
Creatinine	51	62–106 umol/L
Sodium	142	133–146 mmol/L
Potassium	4.4	3.5–5.3 mmol/L
Bicarbonate	25	22–29 mmol/L
Magnesium	0.90	0.7–1.0 mmol/L
Total protein	66	60–80 gm/L
Total bilirubin	5	0–21 umol/L
Alkaline phosphatase	52	40–129 U/L
ALT	17	0–41 U/L
AST	16	0–40 U/L
C Reactive protein	29	0–5 mg/L
RBG	5.3	7.2–11.1 mmol/L
CSF cell count		
Appearance	Colorless, Clear	Clear
Nucleated cell	160	0‐5/uL
RBC	0	0‐2/uL
Lymphocytes	81	40%–80%
Monocyte	18	15%–45%
CSF chemistry		
Protein	1.70	0.15–0.45 gm/L
Glucose	2.06	2.22–3.89 mmol/L
CSF: serum glucose ratio	0.29	2/3 of serum level
Routine urinalysis		
Appearance	Yellow, clear	Yellow
White blood cell	4	0–9 uL
Red blood cell	0	0–9 uL

Abbreviations: ALT, Alanine transaminase; AST, Aspartate transferase; RBG, Random blood glucose.

**FIGURE 1:**
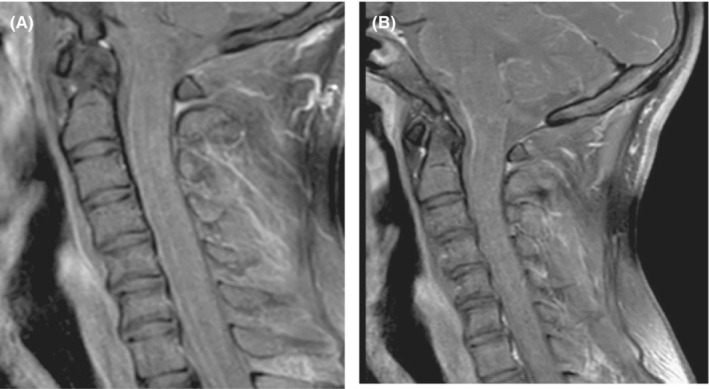
(A and B). MRI Spine Sagittal T1: post‐contrast showing dural thickening of cervical cord area.

**FIGURE 2:**
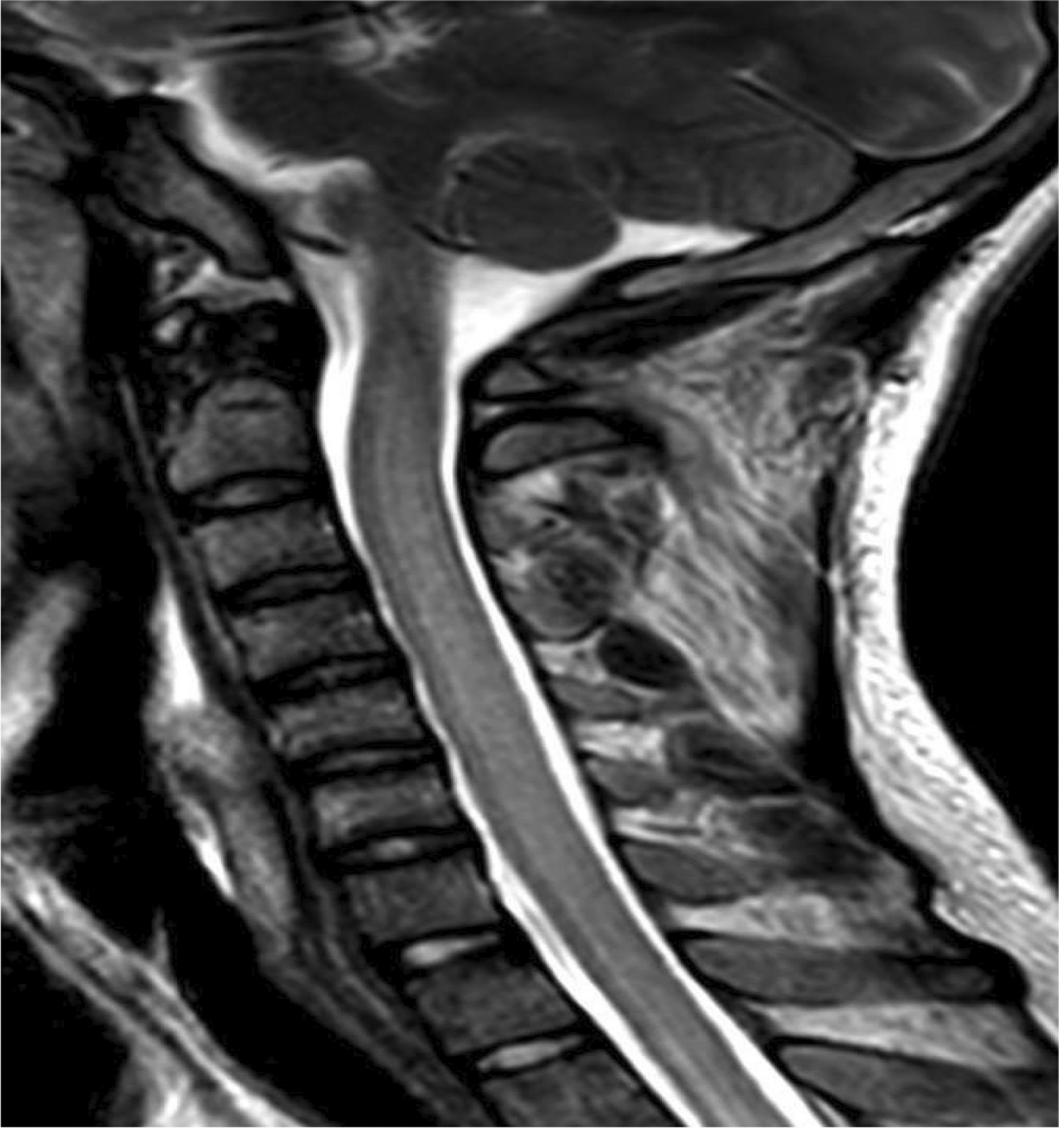
MRI Spine T2 Sagittal: showing cervical cord edema.

CT of the chest did not show any concomitant pulmonary tuberculosis. Within 3 days of his admission, the patient's condition deteriorated. He developed fluctuations in his Glasgow Coma Scale (GCS) 10–12/15, photophobia, marked neck stiffness with worsening of bilateral ascending paresis as indicated by complete loss of power and reflexes in lower limbs, extensor plantar responses followed by a progressive decline in movements of upper limbs. He was empirically started on an anti‐Tuberculosis regimen and steroids through the nasogastric tube route whilst being transferred to the Medical Intensive Care Unit (MICU), sedated, and intubated. To determine whether the treatment was successful, repeat Lumbar Puncture (LP), and MRI were performed. While the LP was unchanged, MRI brain and spine showed a decrease in bilateral leptomeningeal enhancements with abnormally high intramedullary T2 signal intensity of the spinal cord along with diffuse dural thickening (Figure 1A,B) and cord edema (Figure 2).

## 3 OUTCOME AND FOLLOW‐UP

Although there was no laboratory evidence of Mycobacterium tuberculosis bacteria in our patient, he had bilateral paraparesis and urinary retention symptoms, which rapidly progressed to upper limb weakness, areflexia, and signs of meningeal irritation. Based on the presence of these symptoms and a very high clinical suspicion, a diagnosis of TBM was presumed. Additionally, MRI brain showed features suggestive of leptomeningitis and MRI spine showed arachnoiditis, which supported our diagnosis of Probable TB Meningitis. The clinical condition of our patient improved substantially within 2 weeks after being started on Anti Tuberculosis treatment (ATT) – Isoniazid, Rifampicin, Pyrazinamide, and Ethambutol along with steroids. The patient regained full consciousness after 11 days, gained some strength in limbs, and was extubated after minimal ventilator settings. His GCS improved to 15/15, reflexes returned, and he was finally stepped down to the medical ward after 14 days of stay in the MICU. The patient's stay at the ward was extended for 3 more weeks because of ATT drug‐induced hepatitis treatment and monitoring, as well as physical rehabilitation. He was finally discharged for outpatient follow‐up, with a plan of 9 to 12 months of TB treatment.

## 4 DISCUSSION

TB is a significant public health concern that affects both immunocompromised and immunocompetent patients.^4^ In 2021, the World Health Organization (WHO) reported that worldwide, TB is the second major mortality‐causing infectious disease after COVID‐19 pneumonia, and the thirteenth leading cause of death. Additionally, about 1.6 million people died from tuberculous infection in 2021.^5^

TBM is caused by acid‐fast bacilli Mycobacterium tuberculosis which leads to the inflammation of the membranous layers of the brain. It affects just 1% of tuberculosis cases and thus is a rare disorder.^6^, ^7^ Mycobacterium tuberculosis bacilli spread via the bloodstream and lymphatic channels to the central nervous system, thereby involving the brain parenchyma and meninges.^8^ From there on, minute TB granulomas known as rich's focus appear in the brain parenchyma and meninges, and rupture of these lesions results in the release of the rod‐shaped bacteria. This brings about inflammatory changes in the spinal cord, resulting in the development of associated symptoms. TBM causes various spinal complications such as tuberculous radiculomyelitis (TBRM), tuberculoma, granuloma, syringomyelia, vertebral TB, and myelitis. TBRM includes arachnoiditis, a complication associated with TBM. This on average affects those less than 30 years of age and is characterized by sensory paresthesias, root pain, areflexic paraparesis, hypotonia, and bladder malfunction.^8^ Our case was complicated by arachnoiditis that led to the development of paraparesis and urinary retention, as shown by his MRI spine findings. The mortality rate of TBM is very high and underlying risk factors for TBM include associated HIV infection, aging, alcoholism, diabetes mellitus, cancer, immunosuppressive agents, and malnutrition.^9^ Our patient is immunocompetent and HIV‐seronegative and no other primary foci source was found.

TBM initially starts as fever, fatigue, headaches, malaise, vomiting, and eventually progresses into, neck stiffness, radicular pain, paresthesias, bladder dysfunction, personality change, cranial neuropathies, seizures, and stupor occur if no treatment is given, which often leads to coma and death.^10^, ^11^ Hyponatremia, hydrocephalus, stroke, cranial nerve palsies, epileptic seizures, diabetes insipidus, tuberculoma, and TBRM are some of its complications.^11^

It is a bit challenging to confirm TBM and the diagnosis may be made by the physician based on clinical presentation.^12^ Commonly used methods to diagnose TBM include lumbar puncture, analysis of CSF (stain for acid‐fast bacteria and culture), and brain imaging such as CT and MRI. Additionally, newer methods to detect TBM such as NAA assays (nucleic acid amplification) that have high sensitivity (85%–95%) have recently been developed.^12^, ^13^ But there is a lack of widespread availability of these new tests which hampers the ability of physicians to reach the final diagnosis. Classic CSF analysis findings in TBM include the following: lymphocytic leukocytosis, CSF: plasma glucose ratio of less than 50% and increased CSF proteins.^12^, ^13^ It should be noted that both CSF cultures and stain for acid‐fast smear are not highly sensitive, with a sensitivity of about 40%–80% and 20%–40%, respectively. So, there is a possibility of false‐negative results. Therefore, despite the negative test results, the diagnosis of TBM still cannot be ruled out. Hence, in case of strong clinical suspicion and areas with a high prevalence of TBM, the diagnosis of TBM is frequently made on clinical features rather than objective evidence of the pathogen.^12^, ^13^

According to the British Infectious guidelines, TBM is considered to be an extremely emergent disease so it should be treated with empirical anti‐tuberculous treatment as soon as clinical suspicion of this condition is made.^14^ It must be treated approximately for a total of 12 months, beginning with four drugs (isoniazid, rifampicin, pyrazinamide, ethambutol) for 2 months, followed by two drugs (isoniazid, rifampicin) for a minimum of 10 months. Irrespective of the disease severity, adjuvant corticosteroids (prednisolone or dexamethasone) should also be administered to all TBM patients.^14^ In a recent case series study published in October 2022 in China, five patients were followed up who exhibited CNS symptoms with no pathogenic isolation of TB and negative imaging. All the other diagnoses were exhausted, and they were started on a trial of empirical anti‐Tuberculous treatment that significantly improved their condition.^15^ Likewise, our patient also displayed symptoms of paraparesis and areflexia along with worsening of his neurological status. His TB screening was negative and despite that, he showed radical improvement with anti‐tuberculous therapy that resulted in the complete resolution of symptoms, which justifies this point.

## 5 CONCLUSION

Diagnosing TBM can be extremely challenging since it could present with unusual or atypical symptoms that can overlap and coincide with other disease processes. Furthermore, to make the matter worse, the diagnostic test results are delayed and often inconclusive owing to low sensitivity and specificity. Therefore, it is entirely possible for a patient with TBM to present with features like acute paraparesis and urinary retention along with negative TB screening results. In such cases, where physicians highly suspect a diagnosis of TBM, a trial of anti‐tuberculous therapy should be initiated to correct the symptoms as soon as possible without waiting for a confirmatory test result.

## AUTHOR CONTRIBUTIONS

**Jr. Balintona Reynaldo:** Writing – original draft; writing – review and editing. **Memon Noor Illahi:** Writing – original draft; writing – review and editing. **Tarab Iqbal:** Writing – original draft; writing – review and editing. **Sidra M. Nayyar:** Writing – original draft; writing – review and editing. **Abdulqadir J Nashwan:** Writing – original draft; writing – review and editing.

## FUNDING INFORMATION

This study was not funded.

## CONFLICT OF INTEREST STATEMENT

The authors declare that they have no competing interests.

## ETHICS STATEMENT

The article describes a case report. Therefore, no additional permission from our Ethics Committee was required (MRC‐04‐23‐216).

## CONSENT

A written informed consent was obtained from the patient to publish this report in accordance with the journal's patient consent policy.

## Data Availability

All data generated or analyzed during this study are included in this published article.
